# Acceptability and Feasibility of Implementing Accelorometry-Based Activity Monitors and a Linked Web Portal in an Exercise Referral Scheme: Feasibility Randomized Controlled Trial

**DOI:** 10.2196/12374

**Published:** 2019-03-29

**Authors:** Jemma Hawkins, Joanna M Charles, Michelle Edwards, Britt Hallingberg, Linda McConnon, Rhiannon Tudor Edwards, Russell Jago, Mark Kelson, Kelly Morgan, Simon Murphy, Emily J Oliver, Sharon A Simpson, Graham Moore

**Affiliations:** 1 Centre for the Development and Evaluation of Complex Interventions for Public Health Improvement School of Social Sciences Cardiff University Cardiff United Kingdom; 2 Centre for Health Economics and Medicines Evaluation Bangor University Bangor United Kingdom; 3 Division of Population Medicine Cardiff University Cardiff United Kingdom; 4 Centre for Exercise, Nutrition and Health Sciences University of Bristol Bristol United Kingdom; 5 Institute for Data Science and Artificial Intelligence School of Mathematics University of Exeter Exeter United Kingdom; 6 Department of Sport and Exercise Sciences Durham University Durham United Kingdom; 7 Medical Research Council and Chief Scientist Office Social and Public Health Sciences Unit University of Glasgow Glasgow United Kingdom

**Keywords:** exercise referral, physical activity, feasibility studies, wearable technologies, costs, economic evaluation, fitness trackers, activity trackers, exercise, physical activity, accelerometry

## Abstract

**Background:**

Exercise referral schemes (ERSs) are recommended for patients with health conditions or risk factors. Evidence points to the initial effectiveness and cost-effectiveness of such schemes for increasing physical activity, but effects often diminish over time. Techniques such as goal setting, self-monitoring, and personalized feedback may support motivation for physical activity and maintenance of effects. Wearable technologies could provide an opportunity to integrate motivational techniques into exercise schemes. However, little is known about acceptability to exercise referral populations or implementation feasibility within exercise referral services.

**Objective:**

To determine the feasibility and acceptability of implementing an activity-monitoring device within the Welsh National ERS to inform a decision on whether and how to proceed to an effectiveness trial.

**Methods:**

We conducted a feasability randomized controlled trial with embedded mixed-methods process evaluation and an exploratory economic analysis. Adults (N=156) were randomized to intervention (plus usual practice; n=88) or usual practice only (n=68). Usual practice was a 16-week structured exercise program. The intervention group additionally received an accelerometry-based activity monitor (MyWellnessKey) and associated Web platform (MyWellnessCloud). The primary outcomes were predefined progression criteria assessing acceptability and feasibility of the intervention and proposed evaluation. Postal questionnaires were completed at baseline (time 0:T0), 16 weeks (T1), and 12 months after T0 (T2). Routine data were accessed at the same time-points. A subsample of intervention participants and scheme staff were interviewed following the initiation of intervention delivery and at T2.

**Results:**

Participants were on average aged 56.6 (SD 16.3) years and mostly female (101/156, 64.7%) and white (150/156, 96.2%). Only 2 of 5 progression criteria were met; recruitment and randomization methods were acceptable to participants, and contamination was low. However, recruitment and retention rates (11.3% and 67.3%, respectively) fell substantially short of target criteria (20% and 80%, respectively), and disproportionally recruited from the least deprived quintile. Only 57.4% of intervention participants reported receipt of the intervention (below the 80% progression threshold). Less than half reported the intervention to be acceptable at T2. Participant and staff interviews revealed barriers to intervention delivery and engagement related to the device design as well as context-specific technological challenges, all of which made it difficult to integrate the technology into the service. Routinely collected health economic measures had substantial missing data, suggesting that other methods for collecting these should be used in future.

**Conclusions:**

To our knowledge, this is the first study to evaluate short- and long-term feasibility and acceptability of integrating wearable technologies into community-based ERSs. The findings highlight device- and context-specific barriers to doing this in routine practice, with typical exercise referral populations. Key criteria for progression to a full-scale evaluation were not met.

**Trial Registration:**

ISRCTN Registry ISRCTN85785652; http://www.isrctn.com/ISRCTN85785652

## Introduction

### Background

Physical inactivity is a major cause of chronic disease [1]. Addressing inactivity at the population level, and among at-risk groups, is a public health priority [2,3]. Interventions for at-risk groups often center around advice and signposting from primary care [4]. Exercise referral schemes (ERSs) are common [5], usually involving health professional referral to community-based structured exercise programs. Although sustained behavior change is consistently associated with internalized, or autonomous, motivation [6-8], patients often enter such schemes motivated by external sources such as general practitioner advice [9-11]. Thus, according to the self-determination theory [12], a key function for ERSs is supporting transition to autonomous motivation through supporting psychological needs for autonomy (volitional and self-endorsed engagement), competence (personal mastery and effectiveness), and relatedness (meaningful interpersonal connections). Although there is evidence of effectiveness of ERSs in the short-term [11,13-15], studies employing multiple follow-ups consistently demonstrate deteriorating effects over time [13,14], perhaps signaling a need for enhanced motivational support to optimize and maintain effects [11].

In Wales, United Kingdom, the National Exercise Referral Scheme (NERS) was established in 2007, which was implemented in 12 local authorities with embedded randomization to test effectiveness before a Wales-wide rollout [16]. After 12-month follow-up, NERS improved physical activity for patients at risk of coronary heart disease. Mediation analyses indicated that increases in autonomous motivation after scheme exit explained almost half of the between-group differences in physical activity 6 months later [17]. Effects on physical activity fell short of significance for the study population as a whole and among patients referred for mental health reasons [18], whereas process evaluation data highlighted a need for postintervention motivational support to maintain changes in the longer term [19,20].

Growing evidence points to potential motivational effects of behavior change techniques (BCTs) such as goal setting, self-monitoring, and personalized feedback on progress toward goals [21-24]. High-quality goal setting and feedback may support autonomous motivation by enhancing patients’ sense of competence. The increasing popularity of wearable technologies provides opportunities to enhance goal setting and feedback [25], allowing frequent, automatic feedback on goal progress and tailored updating of goals based on achievement [26]. Incorporation of social components such as remote contact with intervention providers and interaction with other service users may support motivation through promoting relatedness to others. Research on wearable technologies in exercise interventions is growing [27] and suggests that use of wearable technologies may increase physical activity levels [28-32]. Evidence, to date, suggests that the combination of wearable activity monitors (eg, pedometers and accelerometers) and accompanying Web components (eg, websites, social media, and cloud technology) can support exercise motivation [25,32-34].

Some research indicates that existing technologies may lack important BCTs, which are known to play a part in increasing physical activity, such as action planning and problem solving [35]. Thus, it is useful to explore the utility of such technologies as additions to physical activity interventions such as ERSs where they may align with or add to BCTs already in use. Furthermore, little is known about the acceptability of wearable technologies to ERS populations, who, due in part to the typically older age of ERS patients [13], may have less technology experience than, or use technology differently to, younger users [36]. Although several studies have examined the perception and use of activity monitors in older populations, this has not been explored specifically within the context of ERSs [37,38]. The role of ERS staff in supporting setup and use of technological interventions, and the feasibility of randomized trial methods to evaluate the supplementation of ERSs with technological interventions, remains to be established [39]. Hence, before a trial of effectiveness, which may fail to deliver definitive answers at great cost should the intervention or evaluation design prove infeasible, feasibility testing is required to investigate the suitability for technological intervention within an ERS context [40-42].

**Table 1:** Summary of progression criteria.

Progression criteria^a^	Measures used	Assessment of whether criteria have been met
PC1. Feasibility to recruit a sufficient proportion of new NERS patients to participate in the trial, with appropriate retention to 12-month follow-up (T2)	Percentage of eligible patients recruited; Percentage of participants retained at T2; Regression models used to identify predictors of loss to follow-up	If >20% of new patients recruited=proceed (*green*); if <5%=full-scale trial unlikely to be feasible (*red*). If 5%-20% (*amber*) of the trial steering committee (TSC) will consider the feasibility of proceeding to a full-scale trial bearing in mind the data presented, representativeness of the sample, and possible steps to increase recruitment; If >80% retained at T2=proceed (*green*), if <60%=full-scale trial unlikely to be feasible (*red*). If 60%-80% (*amber*) of the TSC will consider the feasibility of proceeding based on available data and possible steps to increase retention
PC2a. Trial methodology delivered as intended PC2b. Intervention delivered as intended	Summary statistics for intervention fidelity measures overall and by area; Compliance with study invite processes; Compliance with randomization processes	The TSC will consider the data presented and make a judgement about whether the intervention and trial methodology were delivered as intended
PC3. At least 1 of the 2 intervention components is acceptable to participants	Percentages of participants reporting acceptability of intervention components on self-report questions; Issues regarding acceptability of the intervention components explored in qualitative interviews	The TSC will consider the quantitative and qualitative data and make an overall judgement on whether the intervention is acceptable
PC4. Recruitment and randomization processes acceptable to >50% of recruited participants	Percentages of participants reporting acceptability of recruitment and randomization processes on patient questionnaires; Exploration of understanding and acceptability of recruitment and randomization processes in qualitative interviews	>50% of recruited participants report *agree* or *strongly agree* about the acceptability of recruitment and randomization processes; The TSC will apply discretion in judging whether this criterion has been met or could be addressed to improve acceptability in a full-scale trial
PC5. <20% of control group exposed to the intervention components	Percentage of participants in intervention and control groups who report that they were provided with an MWK^b^ device or accessed the MWC Web platform	<20% of control participants report they have used an MWK device during the study period; <20% of control participants report that they have accessed MWC during study period

^a^PC: progression criteria.

^b^MWK: MyWellnessKey.

In this study, we have described the results of a feasibility randomized controlled trial (RCT) [43] of the implementation of an activity monitor (MyWellnessKey [MWK], Technogym, Italy; [44]) and linked Web portal (MyWellnessCloud [MWC], Technogym, Italy) within the NERS in Wales.

### Objectives

Our primary aim was to assess the feasibility and acceptability of implementing and evaluating the use of MWK activity monitors within the Welsh NERS, to inform decisions on whether to, or how to, proceed to a full trial (see Table 1 for details about the progression criteria). The main objectives were to investigate the following:

1. The feasibility of recruitment and retention.
2. The extent of contamination between arms.
3. The fidelity of intervention and trial methodology.
4. The acceptability of the intervention.
5. The acceptability of randomization.
6. The direction of effect of the intervention on the primary outcome (physical activity) and main hypothesized change mechanism (autonomous motivation).
7. The feasibility of collecting the primary and secondary outcomes, process outcome measures, and economic evaluation methods.

## Methods

### Design

This study was a feasibility RCT, with process evaluation and exploratory economic analysis. Full details of the methodology, including the intervention and measures, are provided in an open-access peer-reviewed study protocol [43].

### Recruitment

Recruitment occurred from January to August 2016 from 8 local authorities in Wales, United Kingdom, purposively selected to provide variation in area characteristics (eg, deprivation, population size, and rurality). Participants were eligible if they (1) were referred to the NERS generic pathway (see Textbox 1) and (2) had the capacity to use the activity monitor (ie, computer access or literacy and an email address). Participants were initially recruited using opportunistic invites from NERS staff [43]. Initial recruitment rates were slower than anticipated. Hence, from week 16 until recruitment closed at week 28, local area co-ordinators forwarded invitation packs containing an information sheet and an expression of interest form (to return to the research team) to all new generic pathway referrals before their initial consultation. On receipt of expression of interest forms, the research team posted recruitment packs to formally recruit interested clients. Participants who returned signed consent forms and completed baseline questionnaires were randomly assigned 1:1 to receive either the intervention (NERS plus MWK) or the control treatment (usual NERS practice) via a computer-generated random allocation sequence created by the South East Wales Trials Unit. During the third month of recruitment, a chance imbalance in allocation of participants to intervention and control groups was noticed (26 control/46 intervention). The randomization algorithm was investigated and was not found to be erroneous; nonetheless, after consultation with the trial steering committee (TSC), it was agreed to amend the randomization to a 2:1 allocation to balance the groups sufficiently to investigate feasibility parameters. The proposed sample size for the study was 286 participants [43]; however, because of slow initial response rates previously mentioned, it was agreed by the TSC and study funder that the study could proceed with a reduced sample of 156 participants. This smaller sample allowed the estimation of feasibility proportions of adherence and retention to within at least 11.5 percentage points either side using 95% CIs (conservatively assuming proportions of 0.5). Owing to the delays in recruitment and study funding constraints, the 16-month follow-up acceleromtery assessment could only be carried out with participants who reached the 16-month point before 31 August 2017; as such only 63.5% of the total sample (99/156) were eligible to complete this final measure. A subsample of intervention participants were recruited to participate in qualitative interviews following randomization. From the individuals who expressed an interest in taking part in the interviews, participants were purposively recruited to provide variation in local authority area, age, and sex.

### Procedure

**Figure 1:**
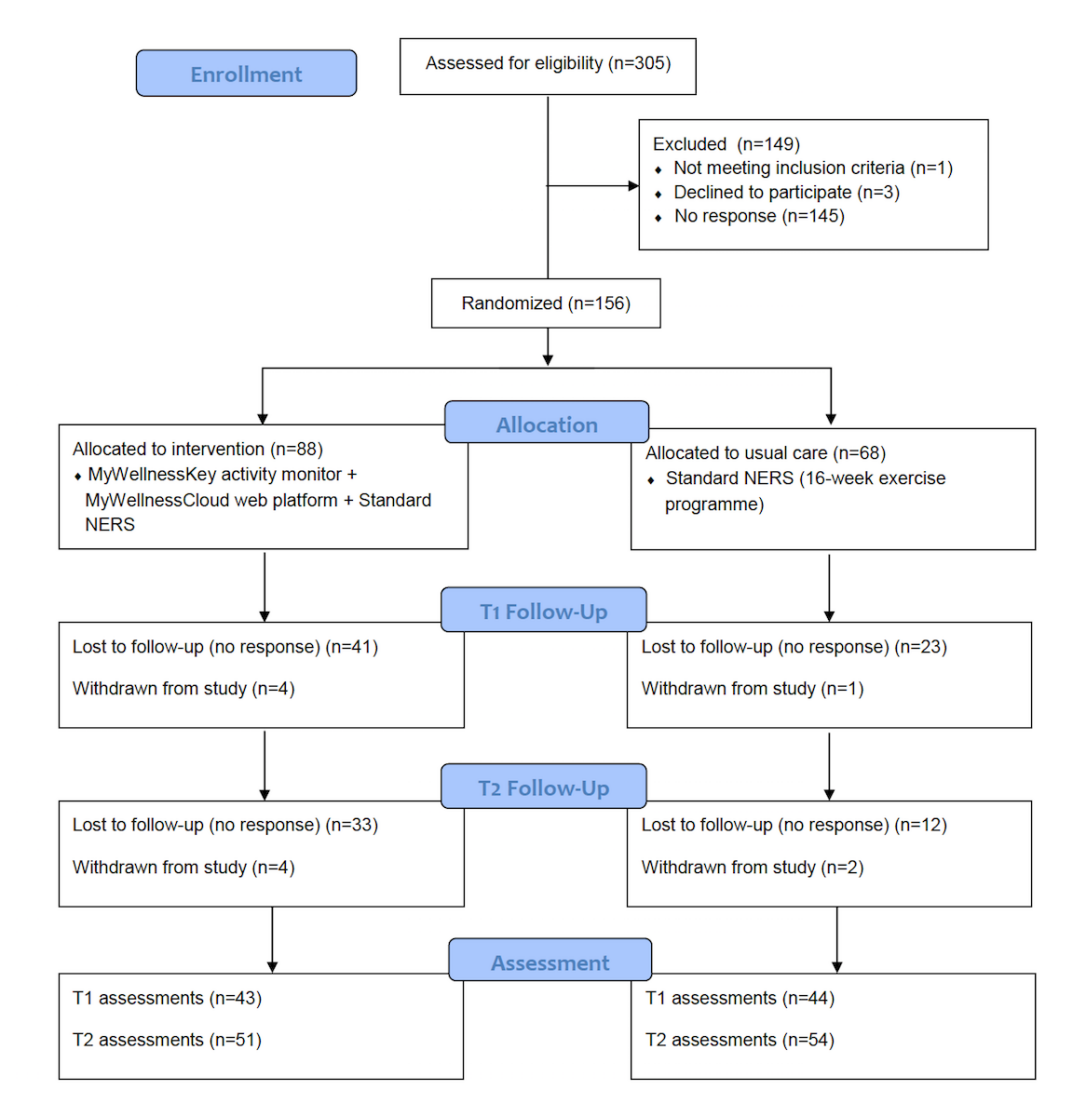
Study flow diagram.

Questionnaire data were collected at baseline (time 0: T0), at the end of the 16-week NERS program (T1) and 12-months postbaseline (T2) via a postal survey. The data collected routinely within NERS were obtained from each of these time points. Semistructured telephone interviews were conducted with a subsample of intervention participants shortly after intervention receipt (n=19) and again at T2 (n=18) and with a sample of NERS exercise professionals (n=11) at the same time points. Participants received full information about the study procedures and the intervention before providing consent, including which intervention was the *intervention of interest*. This study was given favorable ethical opinion for conduct in the National Health Service on 1 December 2015 by the South East Scotland Research Ethics Committee 02 (REF: 189587) and registered with the International Standard Randomized Control Trial Number Register before recruitment. Figure 1 shows the flow of participants through the trial using a CONSORT flow diagram.

**The National Exercise Referral Scheme (NERS) generic pathway referral criteria.**

**For referral into the NERS generic pathway, patients must:**

- be aged 16 years or above;
- be sedentary (defined as not moderately active for 3 times per week or deconditioned through age or inactivity);

**have at least 1 of the following:**

- Raised blood pressure 140/90
- Body mass index >28
- Cholesterol >5.0
- Controlled diabetes or impaired glucose intolerance
- Family history of heart disease or diabetes
- At risk of osteoporosis and/or musculoskeletal pain
- Mild arthritis or poor mobility
- Mild-moderate chronic obstructive pulmonary disorder, asthma, bronchitis, and emphysema
- Mild anxiety, depression, or stress
- Multiple sclerosis

**Figure 2:**
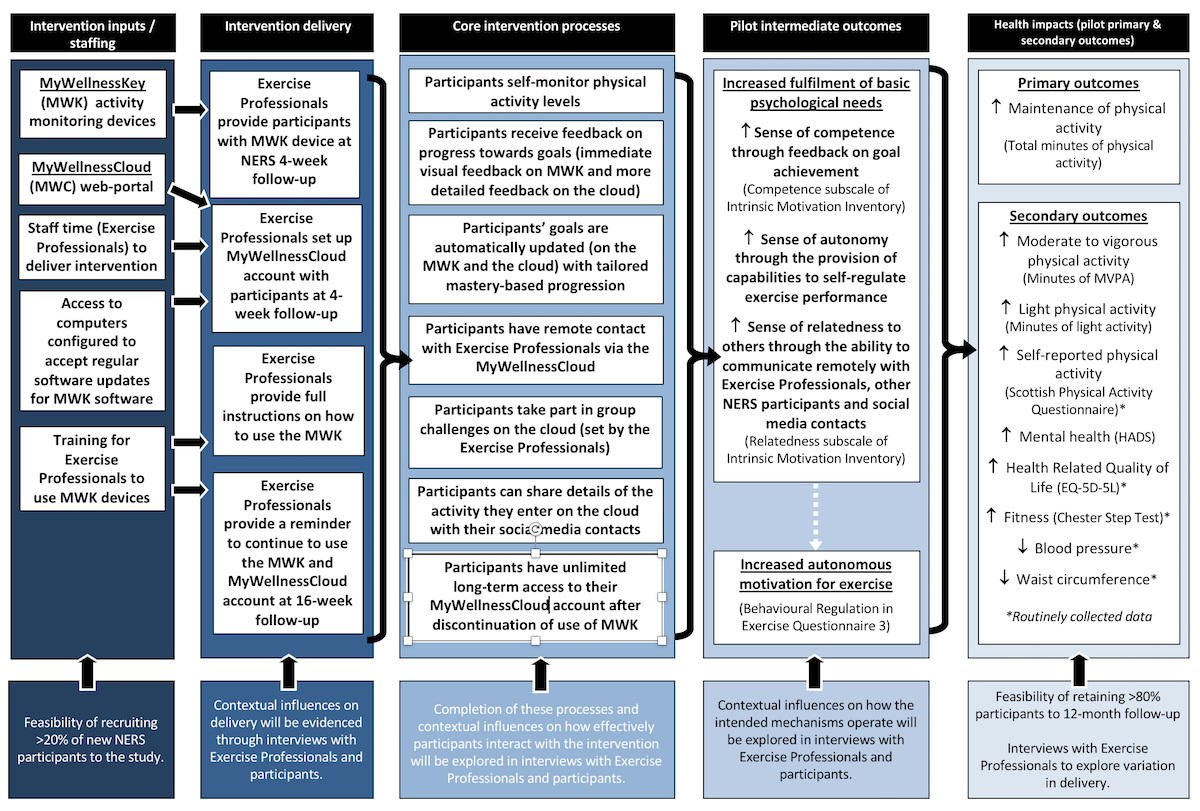
Intervention logic model with study progression criteria.

### Intervention

The intervention was an enhanced exercise referral program, which includes usual care (NERS standard practice; [16]) and an accelorometry-based activity monitor (MWK) and associated Web platform (MWC). The MWK is a uniaxial accelerometer, worn at the hip, with a small screen that provides real-time visual feedback. The MWC allows for provision of more detailed feedback and facilitation of support for behavior change following connection of the MWK to a computer via a Universal Serial Bus (USB). For more information about the MWK and MWC, see the study protocol [43], the intervention logic model (Figure 2), and Multimedia Appendix 1. The MWK devices were chosen for use within the NERS in part because of existing use of other Technogym exercise equipment within the centers in which the scheme is delivered. The NERS exercise professionals followed a protocol to provide intervention participants with an MWK and set up their account on the MWC during their 4-week consultation. Participants could use the MWK and MWC up until their 12-month consultation. Control participants received usual NERS care (a 16-week structured exercise program supported with consultations with an exercise professional at the start, 4 weeks, scheme exit (16 weeks) and 12-month follow-up [16]).

### Measures

A process evaluation was conducted to examine the acceptability and feasibility of intervention and evaluation methods, including intervention delivery and fidelity, potential contamination, and contextual influences. In total, 5 prespecified progression criteria were agreed among the research team and refined after discussion with the TSC. Various quantitative measures, supported by qualitative interview data, were used to assess whether these criteria (see Table 1) were met. This included a traffic light system for certain criteria (red=stop; amber=discuss with TSC whether there is evidence that sufficient improvements can be made to proceed to full trial without another feasibility assessment; and green=proceed). Questions related to acceptability of the intervention were based on key concepts in technology user acceptance such as ease of use and outcome expectations [45,46]. The data sources used to assess the progression criteria are summarized in Table 1. The feasibility of collecting the primary outcome measure for an effectiveness trial (objectively measured physical activity using accelerometry) was examined in a subsample of intervention and control participants using a separate research grade accelerometer (GT3X ActiGraph). Various secondary outcome measures were collected to inform a future trial including self-reported autonomous motivation (Behavioural Regulations in Exercise Questionnaire 3 [BREQ-3]; [47]), psychological need support, anxiety and depression symptoms, physical activity and routinely collected physiological health measures such as blood pressure, body mass index, and fitness (for more information see the protocol paper by Hawkins et al [43]). Measures for the feasibility of an economic evaluation included an adapted Client Service Receipt Inventory (CSRI) to capture client health and social care service use and health-related quality of life measured by the EQ-5D-5L [48]. Copies of the surveys used to collect the self-report measures can be obtained by contacting the corresponding author.

### Data Analysis

#### Quantitative Analysis

The main quantitative analysis involved descriptive summary statistics for each of the study progression criteria (as outlined in Table 1) as follows:

- PC1: *Recruitment:* Percentage of new NERS generic pathway patients recruited to the trial (excluding the first 8 weeks on the assumption that recruitment rates would stabilize over time); and *Retention:* Percentage of participants retained to 12-month follow-up (returning a completed T2 questionnaire).
- PC2: *Trial methods fidelity:* A summary score of adherence to trial recruitment procedure within audio recordings of initial consultations. Recordings were scored according to whether 3 key pieces of essential information about the study were provided, with a total possible score of 3. *Intervention delivery fidelity:* Percentage of intervention participants reporting receipt of the intervention in the T2 questionnaire.
- PC3: Percentages of participants reporting acceptability and use of the MWK and MWC in the T2 questionnaire.
- PC4: Percentages of intervention and control participants reporting understanding and acceptability of the randomization process in the T1 questionnaire.
- PC5: Percentages of intervention and control participants reporting exposure to the intervention (MWK and MWC) during the study in the T2 questionnaire.

Regression models were used to estimate direction of intervention effects on accelerometer-measured physical activity (16 months) and autonomous motivation (16 weeks and 12 months) as measured with the BREQ-3 [47]. Accelerometer data were processed using standard procedures; periods of ≥60 min of zero counts were recorded as *nonwear time* and removed. Participants were included in the analysis if they provided ≥3 valid days of 500 min of data between 6 am and 11 pm; this value, which is at the lower end of thresholds typically used in the literature [49], was selected to maximize representativeness of the sample within the sedentary population under study. Threshold values for mean minutes of different intensity activity were based on Troiano et al [50]. Sedentary time was estimated based on a cut-point of less than 100 counts per minute, and mean sedentary minutes per day were derived. Linear regression models were fitted for each physical activity outcome controlling for age, gender, baseline self-reported physical activity, and allocation arm. Owing to skewness, mean minutes of moderate to vigorous activity were transformed using a square root transformation. For autonomous motivation, models were fitted for the Relative Autonomy Index (RAI; [47]) score of the BREQ-3 controlling for baseline RAI, age, gender, and number of referral reasons.

#### Health Economics Analysis

The economic analysis was conducted from a public sector multiagency perspective. Completeness and availability of data using descriptive statistics was used to examine the feasibility of calculating cost-effectiveness alongside a future RCT. Costs of the intervention were calculated by revisiting and revising the costing methodology used in previous economic analysis of the NERS [51]. Quality adjusted life years (QALYs) were calculated from the EQ-5D-5L using the area under the curve method [52]. To address uncertainty in outcomes (QALYs) and costs (service use), bootstrapping (5000 replications) was used to produce 95% CIs around differences in costs and outcomes. Further details can be found in the study protocol [43].

#### Qualitative Analysis

Qualitative data were transcribed verbatim and organized and coded into a thematic framework using NVivo 11 software (QSR International). The analytic approach incorporated a deductive and inductive approach [53] with data coded using an *a priori* coding scheme of categories aligning with the progression criteria as a means of organizing the data for subsequent interpretation. An element of flexibility was maintained to account for emergence of any new and unexpected themes.

#### Triangulation

Quantitative and qualitative data were analyzed in isolation with individuals responsible for each analysis blind to the other (eg, statistical analysis conducted by members of the team who were not present for management group meetings where qualitative findings were discussed). On completion of all analyses, the data were then brought together; qualitative data were used to provide further detail and highlight possible explanations for the quantitative findings. Data are organized thematically, drawing on both quantitative and qualitative data sets to provide insights into quantitative feasibility metrics and qualitative insights into barriers and facilitators from multiple perspectives, before an overall picture of progression criteria and decision making on proceeding is presented.

## Results

### Baseline Characteristics

Participants (N=156) were aged 56.6 (SD 16.3) years and mostly female (101/156, 64.7%) and white (Table 2). There was substantial socioeconomic bias in uptake, with more than half of recruited participants residing in the least deprived quintile of Wales.

**Table 2:** Baseline (T0) participant characteristics.

Characteristics		Intervention (N=88)	Control (N=68)	Total (N=156)
Age (years), mean (SD)		55.1 (17.6)	58.5 (14.4)	56.6 (16.3)
Female, n (%)		51 (60)	50 (74)	101 (64.7)
White, n (%)		84 (96)	66 (97)	150 (96.2)
**Welsh Index of Multiple Deprivation, n^a^** **(%)**				
	1–most deprived	2 (2)	0 (0)	2 (1.3)
	2	3 (3)	5 (8)	8 (5.2)
	3	8 (9)	2 (3)	10 (6.5)
	4	22 (25)	24 (36)	46 (29.9)
	5–least deprived	52 (60)	36 (54)	88 (57.1)
**Income, n^b^** **(%)**				
	Less than £5000/year	4 (5)	4 (7)	8 (5.5)
	£5000-£9999	7 (8)	11 (18)	18 (12.4)
	£10,000-£15,499	22 (27)	15 (24)	37 (25.5)
	£15,500-£20,999	18 (22)	10 (16)	28 (19.3)
	£21,000-£30,999	12 (15)	10 (16)	22 (15.2)
	£31,000-£50,999	16 (19)	7 (11)	23 (15.9)
	£51,000 and more	4 (5)	5 (8)	9 (6.2)

^a^A total of 2 participants did not complete this measure, 1 from intervention and 1 from control.

^b^A total of 11 participants did not complete this measure, 5 from intervention and 6 from control.

### Recruitment and Retention to the Trial, Contamination, and Acceptability of Randomization (Progression Criteria 1, 2, 4, and 5)

#### Recruitment

Recruitment fell substantially short of the target of 20% (11.28% [156/1382] of new NERS patients were recruited). After excluding the first 8 weeks, this figure remained similar at 10.99% (111/1010), with 9.1% (31/339) recruitment achieved in the final 8 weeks. Only 6 of 26 (23%) staff provided the audio recordings (N=12) required for assessment of fidelity to the recruitment process. In total, 5 recordings scored 0, with the highest score achieved being 1.75 (out of a total of 3); key information was frequently omitted, which participants might require to make a decision about being contacted by the research team, such as the intervention involving an activity monitoring device or that using it required access to a computer. Qualitative interviews with staff provided explanations for limited adherence to recruitment procedures, including that it was easy to forget to mention the study because it was not part of usual practice, with parts of the procedure often omitted (confirmed by recordings):

It’s quite difficult, ’cause sometimes even during the consultations, you’re kind of talking through it, and ’cause we’re on auto pilot, when it comes to asking [about their interest in joining the study], I didn’t always remember to do it.

EP22-T2

If there is someone who is referred and they can hardly move and they’re old and they don’t have a computer I don’t see the point even to talk about it.

EP71-T1

#### Retention and Attrition

A retention rate below the target of 80% was achieved at T2 (105/156; 67.3%). Univariate logistic regression (Table 3) explored potential predictors of follow-up. Retention in the control group of 75% (51/68) was achieved versus 61% (54/88) in the intervention group (odds ratio (OR) 0.53 (95% CI 0.26 to 1.06).

In follow-up phone calls with the 21 participants who did not respond to the T2 questionnaire, 9 reported that disengagement from the NERS was the reason for not completing study questionnaires and 5 cited issues with the MWK as their reason. Staff perceptions of barriers to recruitment and retention also focused on technological problems with the MWK such as lack of internet access or use of another activity monitor and typical disengagement with the NERS:

Yeah. there were a couple of older clients who weren’t computer literate, and there was one or two who said they didn’t have access to any sort of computing.

EP51-T2

We have three attempts to get back in touch with [non-engaging] clients, like three phone calls and a letter, and if they don’t respond, I can’t harass them.

EP82-T2

**Table 3:** Predictors of loss to follow-up.

Variable	Odds ratio (95% CI)
Intervention group (N=156)	0.53 (0.26-1.06)
Female (N=156)	1.29(0.65-2.58
Most affluent (N=154)	0.62 (0.31-1.25)
Baseline motivation (N=129)	1.01 (0.99-1.02)
Baseline physical activity (N=134)	0.97 (0.61-1.54)
Multiple referral reasons (N=134)	0.73 (0.32-1.71)

#### Contamination and Fidelity to Random Allocation

At T2, 10% of control participants (5/51) reported exposure to 1 of the 2 intervention components, whereas 22% of responding intervention participants (12/54) reported that they had not been given an MWK during the study. The proportion of participants who reported using non-MWK activity monitors in the last 12 months was similar in both the control group (12/51, 24%) and intervention group (13/54, 24%). One individual from the control group reported that their decision to use another activity monitor was influenced by participation in the trial. In total, 2 intervention participants reported that they had used another device because of problems they had with the MWK. Staff interviews confirmed the occurrence of contamination, with 1 member reporting giving an MWK to a control participant and 3 reporting advising control participants on how they could access an MWK elsewhere.

#### Acceptability of Randomization

At T1, 93% (79/85) of participants reported understanding the use of a control group, whereas 84% (72/86) either agreed or strongly agreed that it was acceptable to only give the MWK to half of the participants and 96% (82/86) either agreed or strongly agreed that it was acceptable that the MWK was given to half of the participants at random. Despite high acceptability of randomization in quantitative data, the staff reported that some clients were disappointed by control group allocation. Although the use of other devices was similar across arms, interview data from intervention participants suggested that some might have bought a different activity monitor if they had been allocated to the control group.

### Feasibility and Fidelity of Intervention Delivery (Progression Criterion 2)

At T2, 57% of intervention participants (31/54) reported that they had received an MWK during the study, which was below the criterion threshold of 80%. Of those who received the intervention and participated in the T1 questionnaire (n=40), 94% (34/36) stated that their exercise professional provided them with information on how to use the MWK and MWC. Of these, 35% (12/34) reported they received sufficient information on the MWK only, whereas 62% (21/34) received enough information about both the MWK and MWC. Qualitative interviews with staff highlighted a number of issues with Information Technology (IT) and time constraints, which were perceived to have hampered the setup process:

I’m aware that some had issues with our MWKs. I know we had issues with setting up the MWKs and with our IT...And also for me, as an instructor, it took a bit of time to set them up.

EP42-T2

Most of these issues were linked to either the MWK device or the delivery context (eg, issues with USB devices and IT system security), with fewer being staff-specific (eg, having not attended training or low IT literacy):

Because our laptops are encrypted there sometimes can be a bit of an issue with trying to open up the MWC. Also, we couldn’t actually download the software to assign MWKs to people [because of firewalls] so the IT department had to over-ride it for us.

EP61-T1

In some areas, the staff made attempts to overcome IT issues by using their own laptops or helping participants to set themselves up with the MWK at home:

Well I charge the MWK and I give it to them and I give them the instructions to do it at home [...] I ask them beforehand if they’re computer literate and would they be happy to do it themselves.

EP71-T1

### Intervention Acceptability (Progression Criterion 3)

Use of both intervention components was reported by approximately half of the participants, though in both cases this diminished over time. At T2, 57% of intervention participants (31/54) reported using the MWK at some point during the study. However, only 8% (4/49) had used it in the past month. Just under half reported using the MWC at some point during the study, with only 6% (3/47) having used the MWC within the past month. See Multimedia Appendix 2 for a summary of mean scores for acceptability and usage questions. Patient interviews suggested that some engaged with the device initially but stopped owing to a combination of device malfunctions and the novelty factor wearing off:

Over a few days, I did quite a lot of exercise and nothing was registered on there. So to be honest with you, I lost a lot of confidence in it. I explained it to my instructor, and he said just carry on with the exercise anyway. So I haven’t really used it because nothing was registering.

BL130-T2

Other factors influencing engagement included lack of access to a computer and/or internet, poor IT literacy, and technical issues with charging and syncing the device, sometimes highlighting reliance on a relative or the instructor to support continued use:

I needed technological help to explain what had to be done really, and I wasn’t altogether the most brilliant at this technology on the system, so I had help from my instructor about that.

PE088-T2

The proportion of patients rating the components as easy to use was 49% (21/43) for the MWK and 33% (14/42) for the MWC. Qualitative data highlighted challenges in understanding how the device worked, wearability issues, and not understanding how to use the MWC:

I don’t think it’s the best design to be perfectly honest, it’s difficult to attach to your clothing, I think perhaps for a chap it’s a little bit easier because they generally wear something with a waistband but women, especially in the summer time often don’t, and I think I’m going to struggle in the summer when I’m wearing dresses to find somewhere to put it where it’s horizontal.

AN080-T1

The proportion of patients reporting that they would use either device in future if they could was 37% (17/46) for the MWK and only 15% (7/46) for the MWC. In the qualitative interviews, participants suggested that they would be more likely to use the intervention in the future if it was easier to understand, technical issues were addressed, and it had better wearability:

If it was easier to charge, ’cause the battery kept going, and if it was easier to wear. Being a girl...if I had a dress on for instance, there was nowhere to put it...if I didn’t have a pocket or anything like that, then there was nowhere to actually wear it. So if it’d been like on a wristband or something similar, then I probably would have worn it more, I would have just left it on the top with my watch and put it on every day and I’d probably still be using it.

MO106-T2

A small majority (26/46, 57%) reported that the device met their expectations in terms of motivating them to be physically active; qualitative data suggest that the reasons it did not meet expectations were linked to the issues reported above:

It was beyond what I was hoping for, I’ve got to be honest. I enjoyed that you could manually enter [on the MWC] if you were doing individual weights and weight machines...or if you were in the garden, and these sorts of things, so I wasn’t expecting that.

PE154-T2

I was hoping it’d be more like a Fitbit, ’cause Fitbits are generally quite easy. But it seemed to be a little bit more complicated than that, I thought, or needed more attention than the Fitbit.

BR148-T2

### Direction of Effect on Physical Activity and Hypothesized Change Processes

Of the 99 participants (53 control and 46 intervention participants) eligible to provide accelerometer data, 54% (53/99) consented to do so; and 89% of consenting participants provided valid useable data (26/30 control and 21/23 intervention). Of the 6 people who did not provide valid data, 3 did not record sufficient data to meet validity thresholds and 3 did not return the accelerometer. As displayed in Table 4, trends were in the direction of a positive outcome only for sedentary behavior, though with wide CIs for all outcomes. For autonomous motivation, trends were in the direction of a negative outcome at both 16 weeks and 52 weeks (Table 4).

### Feasibility of Conducting an Economic Evaluation

#### Response Rates and Level of Completion

Overall, 156 participants completed baseline economic measures, 85 participants at T1 and 105 participants at T2. Missing data ranged from 0% to 22% (see Multimedia Appendix 3). The EQ-5D (5L) [54], which was obtained from the NERS database, had the largest proportion of missing data of the 2 economic measures. The limited missing data from the CSRI within the study questionnaire show that it is feasible to collect health and social care service use from patients in a future trial. There were limited missing data for measures of productivity losses, ranging from 0% to 18% (see Multimedia Appendix 3).

As shown in Table 5, a total of 25 cases were available for between group comparison of QALYs, and there were 105 cases available for between group comparison of total service use. Service use was lower in the control group, with a significant difference between groups of £386 (including cost of intervention), whereas there was a nonsignificant difference in QALYs between groups of 0.07 QALYs in the opposite direction, equating to 26 days.

**Table 4:** Direction of intervention effects on physical activity and autonomous motivation.

Variable		Coefficient (95% CI)
Moderate to vigorous physical activity (N=45)		−0.23 (−1.54 to 1.09)
Volume of physical activity (N=45)		−1.20 (−82.42 to 80.0)
Sedentary behavior (N=45)		−18.5 (−81.99 to 44.91)
**Autonomous motivation**		
	16 weeks (N=74)	−3.63 (−14.24 to 6.97)
	52 weeks (N=95)	−4.14 (−13.47 to 5.19)

**Table 5:** Mean quality adjusted life years (QALYs) at 52-week follow-up (T2) by group (mean QALYs at follow-up and 5000 bootstrapped 95% CIs all rounded to 2 decimal places). Mean total service use costs at 52-week follow-up (T2) including the cost of the intervention (mean total service use costs at follow-up and 5000 bootstrapped 95% CIs all rounded to 2 decimal places).

Variable	Intervention group		Control group		Difference between groups (5000 bootstrapped 95% CI)
	n	Mean (SD)	n	Mean (SD)	
QALYs over one year (T2)	11	0.71 (.09)	14	0.78 (.14)	0.07 (0.016 to 0.02)
Total service use costs at T2 including cost of intervention	54	£870 (1332.66)	51	£484 (1230.27)	£386 (35.80 to 452.53)

**Table 6:** Costs of delivering the National Exercise Referral Scheme (NERS) with MyWellnessKey (MWK) as part of the feasibility trial.

Annual NERS operational costs 2016-2017		Total (£)^a^
**National costs paid by the Welsh government**		
	Consultant	2384
	Physical activity specialist (Grade 8a)	10,684
	Administrative support	2530
	Health improvement coordinator	1392
	Meeting costs	300
	Exercise professionals (91.5 Whole Time Equivalent [WTE])	2,631,385
	Coordination and office costs (eg, printing and stationary) for all 22 local authorities	71,848
	Training	64,495
	Travel	80,547
**Joint national and local costs**		
	Co-ordinator salary (23 WTE) funding is split between local authorities (£368,438) and the Welsh Government (£478,319)	846,757
**Local authority costs**		
	Staff management	75,000
	Promotional material	22,000
	Room hire (no charge as covered by session costs)	0
	Attending conferences	2200
	Total NERS annual operating costs (without MWK)	3,811,522
	Participants in NERS^b^	15,626
	Cost per participant	244
**Additional costs related to MWK**		
	Cost of MWK activity monitor device (based on 88 units purchased for the trial intervention group)	3960 (£45 per monitor×88)
	Cost of MWC annual license fee (professional Web cloud) including Value-Added Tax	3360
	Total MWK operating costs	7320
	Participants in receipt of MWK as part of the trial	88
	Cost per participant for MWK	83
	Total cost per participants for NERS with MWK^c^	327

^a^Costs rounded to the nearest pound (£).

^b^Participants in the NERS based on 15,470 individuals who took up the NERS program from September 2016 to August 2017 including the 156 participants taking part in the trial (intervention n=88, control n=68).

^c^Calculation—total annual operational cost per participant and total cost per participant for MWK.

**Table 7:** Summary of results of progression criteria assessment.

Progression criteria	Results	Criteria met or not
PC1. Feasibility to recruit a sufficient proportion of NERS patients, with appropriate retention rates to T2	11.3% of new NERS patients recruited; 67.3% of study participants retained at T2; No significant predictors of loss to follow-up identified	Not met
PC2a. Trial methodology delivered as intended; PC2b. Intervention delivered as intended	57.4% of intervention participants reported having received the intervention; 35.3% of intervention participants received sufficient information on how to use the MWK; 61.8% received sufficient information for both the MWK and MWC	Not met
PC3. At least 1 of the 2 intervention components is acceptable to participants	49% (MWK) and 33% (MWC) of participants reported the intervention components as easy to use; 37% (MWK) and 15% (MWC) of participants reported that they would use the intervention components in the future; Interview data highlighted challenges in IT and device literacy, technical issues, wearability, and computer access	Not met
PC4. Recruitment and randomization processes acceptable to >50% of recruited participants	92.9% of participants reported understanding the use of a control group; 83.7% of participants agreed that it was acceptable to only give the intervention to half of participants and 95.4% agreed that random allocation was acceptable	Met
PC5. <20% of control group exposed to the intervention components	9.3% of control group participants reported exposure to one of the 2 intervention components	Met

#### Costs of the Intervention

The costs of NERS are presented for the cost year 2016-2017. Under a delivery framework in which the intervention was absorbed into existing staff roles, the only additional cost of the intervention was the cost of the MWK devices and the annual licence fee for the MWC, which combined with the usual NERS delivery totals £3,818,842 equating to £327 per person based on the 88 intervention participants in this study (Table 6).

As part of a sensitivity analysis, the costs of the NERS were varied using the retail price of the MWK device of £90, rather than the lower price of £45 that they were purchased at. Multimedia Appendix 4 shows the cost of the NERS with MWK when varying the price of the MWK device was £3,822,802; this equates to a cost per person of £352, based on the 88 intervention participants in this study.

#### Willingness to Pay for Device

In the T2 questionnaire, participants (n=54) responded that they were willing to pay a mean of £29 to use the device during the NERS, reducing to £23 to keep the device afterward. Participants reported willingness to pay as much as £110 (n=2) for the device; however, the minimum amount participants were willing to pay was £0 (n=11).

### Progression Criteria for a Full-Scale Trial

Only 2 of the 5 criteria for progressing to a full-scale evaluation were met (see Table 7). Recruitment and retention rates were within the *amber* progression zone, indicating the need for TSC discussion about the feasibility of proceeding, taking into account the data and feedback presented (Table 1). Although the qualitative data provided some insight into barriers to recruitment and retention and possible steps to improve this, it was felt that these issues could not be addressed sufficiently to justify progression without further feasibility work. In addition, the intervention acceptability data did not meet the criterion threshold and there were issues with fidelity of intervention delivery and compliance with randomization processes. In discussion with the TSC, it was agreed that an effectiveness trial would not be feasible given the issues faced with recruitment and retention and the feasibility and acceptability issues related to implementation of the intervention in practice.

## Discussion

### Principal Findings and Comparisons With Prior Work

This study identified a range of challenges in integrating accelerometer-based wearable technologies into an existing community-based exercise referral program and evaluating this using RCT methodology. There were a number of issues with recruitment and retention of participants and intervention implementation. High attrition, particularly in intervention groups, is common in technological and Web-based health intervention research [55], reflecting challenges in maintaining interest in utilizing such technologies; nonusage attrition often increases steadily over time, with disengagement over time typically observed [55,56]. In this study, less than 10% of intervention participants were still engaging with the device in the final month of the study.

Acceptability of the activity monitors to the ERS population was mixed, with various barriers to use identified. This included wearability and technical problems (eg, difficulty connecting the device to computers and accuracy problems with activity tracking). Comfort and practicality of device wearing has been commonly raised [57,58], particularly among females and within older populations (demographics who make up a large proportion of ERS participants). How a device looks and how secure it is when it is attached are key issues associated with device usage [59], with comfort and wearability closely tied to a device’s perceived ease of use and individuals’ decision to use [60]. Technical problems are also commonly reported as barriers to engagement [61,62], with individuals reporting issues including device malfunction [63], problems with accuracy of devices for tracking activities [57], and difficulties accessing feedback on the activity tracked [58]. In this study, some participants cited limited IT literacy as a barrier to engagement, with some noting their age as a factor in their unfamiliarity with such technology, consistent with previous evaluations in older populations. Possibly linked to this, engagement and acceptability ratings for the MWC Web platform were lower than those for the device itself.

Although some studies suggest that similar technologies are both acceptable and feasible to use with adults aged up to 75 years, some have cited difficulties with software installation and the use of associated websites [63]. New and emerging technologies have been perceived as outside the comfort zone of older populations, with an added need to learn the *language* of a device being a barrier to use [59]. Although research on wearable technologies has commonly focused on younger populations, evaluations in populations similar to that of the NERS have identified a need for more extensive support for participants in setup and troubleshooting devices [64], with some implementing high levels of training and troubleshooting within initial months of intervention delivery [38]. Although this study focused on a low-cost delivery model, which integrated this support into roles of existing scheme staff, it may be that such a model is appropriate only where interventions occur in a context of relatively high IT literacy populations. Challenges were perhaps exacerbated by some professionals’ lack of buy-in, with professionals describing being distracted by more pressing concerns during time with patients, which limited their ability or willingness to recruit to the study.

Long-term physical activity assessments revealed challenges in use of accelerometers as an outcome measure in this community-based intervention, including low response rates. This study was not intended to assess effectiveness, given its size and limited power. For measures of motivation and physical activity (although not sedentary behavior), directions of effect pointed toward negative impacts, although with wide CIs either side of 0. It is common practice to provide between group comparisons for primary outcomes within feasibility trials to demonstrate that a planned analysis approach is likely to be feasible. However, as feasibility studies are small and underpowered, interventions commonly continue to be refined after a feasibility study and as samples are likely to be unrepresentative of those recruited to a larger trial, such estimates are unlikely to provide meaningful estimates of the likely effect of an intervention. Hence, such data ought to be interpreted with extreme caution. There were substantial missing data from the health economic measures that were routinely collected within the NERS, providing difficulty with conducting an economic evaluation using these methods of data collection. Future work within this population should collect economic data through other self-report methods.

### Strengths and Limitations

This study has a number of strengths and limitations. To our knowledge, it is the first to evaluate the use of wearables in an exercise referral population and to explore issues associated with embedding and evaluating technologies within an established community-based intervention. It employed a robust study design including a mixed-methods process evaluation at multiple time points to measure and understand engagement, acceptability, and usability of the intervention alongside piloting measures for an effectiveness study. However, although study sites were purposively sampled to provide a range in levels of area deprivation, the recruited sample was skewed toward a more affluent population, perhaps owing to the more affluent study sites having larger populations or reflecting differences in engagement with activity monitor interventions between socioeconomic groups. The study sample size was originally planned to provide power to detect an effect on the hypothesized mediator, autonomous motivation. However, owing to lower than expected recruitment, sample size targets were revised and hence analyses lack power. Finally, the study evaluated a commercially available device, which is no longer being manufactured. Although there are newer technologies available that overcome some of the issues identified with the MWK (lack of Bluetooth connectivity and issue of wearing at the hip), it is not clear whether these offer the same range of opportunities for behavior change support—particularly from exercise intervention providers.

Nevertheless, these findings offer a number of important insights for future studies. First, although the potential efficiency gains of integrating support and troubleshooting roles into those of existing staff may be appealing, where working with populations with more limited IT skills, additional investment in external support may be required. This is perhaps particularly the case where interventions operate in uncontrolled real-world settings, where professionals serve a large number of clients, as in the NERS, and hence cannot commit much time to supporting engagement with the intervention. Clearly, an introduction of additional technical support components would drive up intervention costs, meaning that effects would perhaps need to be relatively large to justify this investment. Second, recruiting participants to an RCT via routine consultations held by exercise professionals proved challenging; a future full-scale evaluation of similar interventions would require feasible alternative recruitment mechanisms to be established. Finally, as many issues were raised by participants related to the specifics of the MWK devices, the extent to which findings are generalizable to other wearable technology interventions is not always clear. The rapidly evolving nature of wearables and similar technologies presents challenges for efficient and timely evaluation, and RCT methods have been suggested as too slow an approach compared with other more efficient methodologies when evaluating technologies which become out of date during the study period [27].

### Conclusions

This study provided an examination of the short- and long-term feasibility and acceptability of integrating wearable technologies into existing community-based ERSs, highlighting some of the possible device- and context-specific barriers. Key criteria for progression to a full-scale evaluation were not met owing to difficulties integrating the technology into routine practice, facilitating uptake by patients, and in methodological challenges relating to the collection of long-term follow-up data. This study demonstrated the importance of investing small amounts of research funding in feasibility assessment before conducting expensive full-scale effectiveness evaluation, which may fail to be fully executed because of problems with implementing the intervention or evaluation methodology.

## Appendix

Multimedia Appendix 1 Screenshots of the MyWellnessCloud Web platform.

Multimedia Appendix 2 Mean scores for usage and acceptability of intervention components.

Multimedia Appendix 3 Frequencies and percentage of missing data for whole sample at each time point.

Multimedia Appendix 4 Costs of delivering the National Exercise Referral Scheme (NERS) with the MyWellnessKey (MWK) as part of the feasibility trial varying unit cost of the MWK device.

## Abbreviations

BCTs: behavior change techniques
BREQ: Behavioural Regulations in Exercise Questionnaire
CSRI: Client Service Receipt Inventory
ERS: exercise referral scheme
MWC: MyWellnessCloud
MWK: MyWellnessKey
NERS: National Exercise Referral Scheme
PC: progression criteria
QALYs: quality adjusted life years
RAI: Relative Autonomy Index
RCT: randomized controlled trial
TSC: trial steering committee
