# Supplementary material for: Acceptability and Feasibility of Implementing Accelorometry-Based Activity Monitors and a Linked Web Portal in an Exercise Referral Scheme: Feasibility Randomized Controlled Trial
Source: J Med Internet Res. 2019 Mar 29;21(3):e12374. doi: 10.2196/12374 (PMC6460312; doi:10.2196/12374)
Supplement: Multimedia Appendix 1 [file jmir_v21i3e12374_app1.pdf]

Screenshots of the MyWellnessCloud web platform.

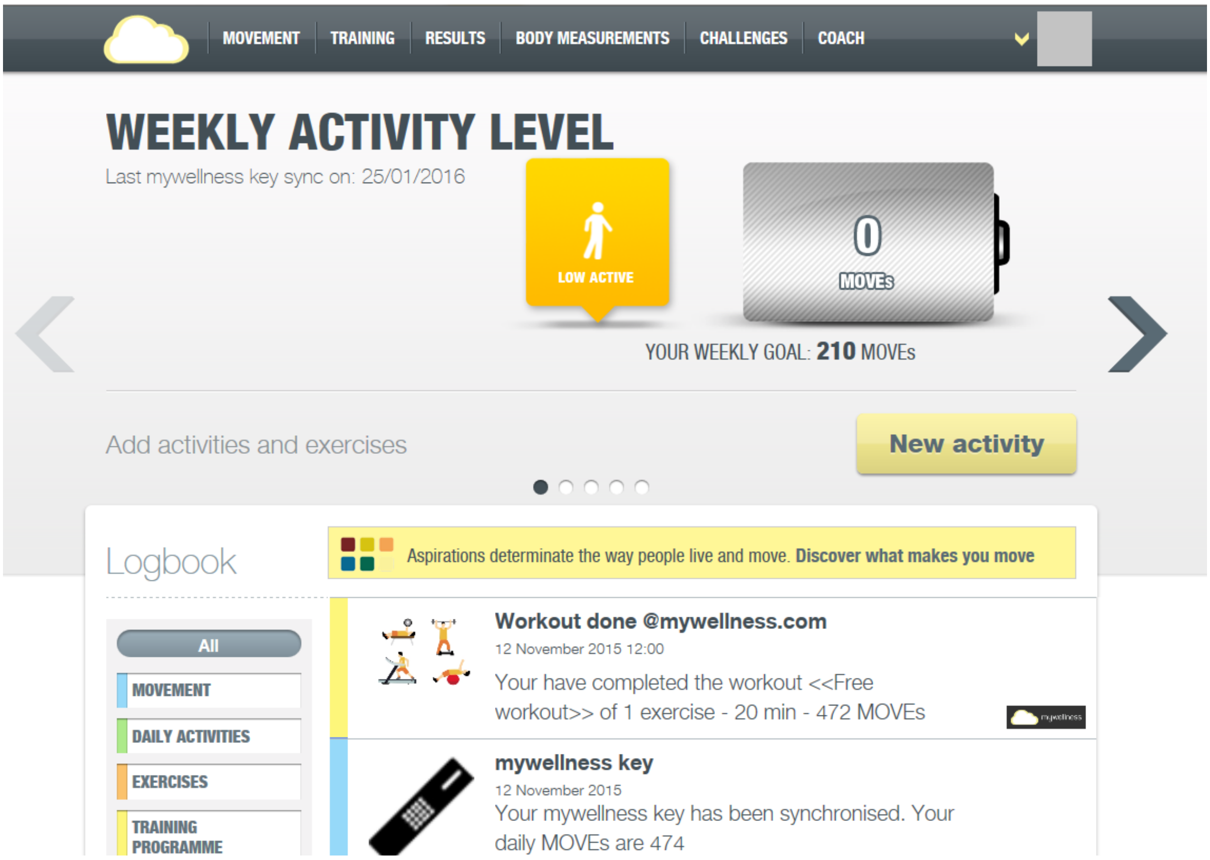

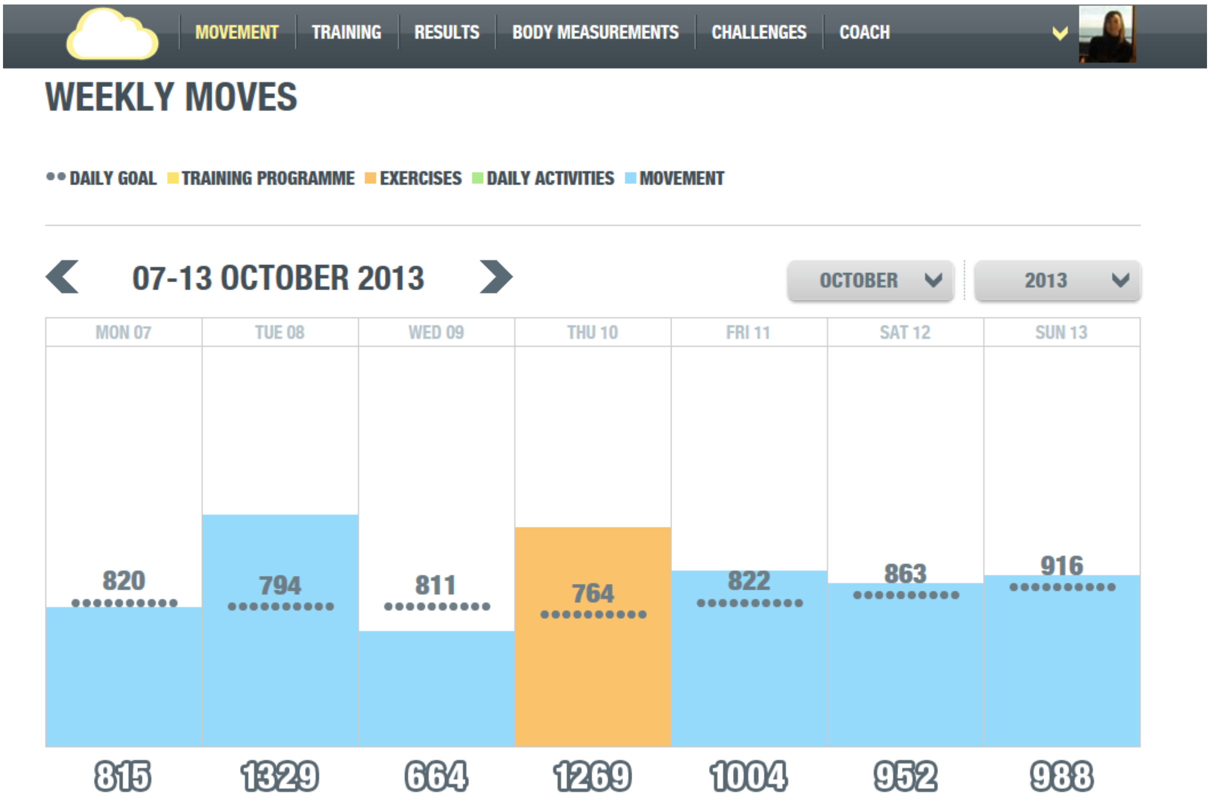

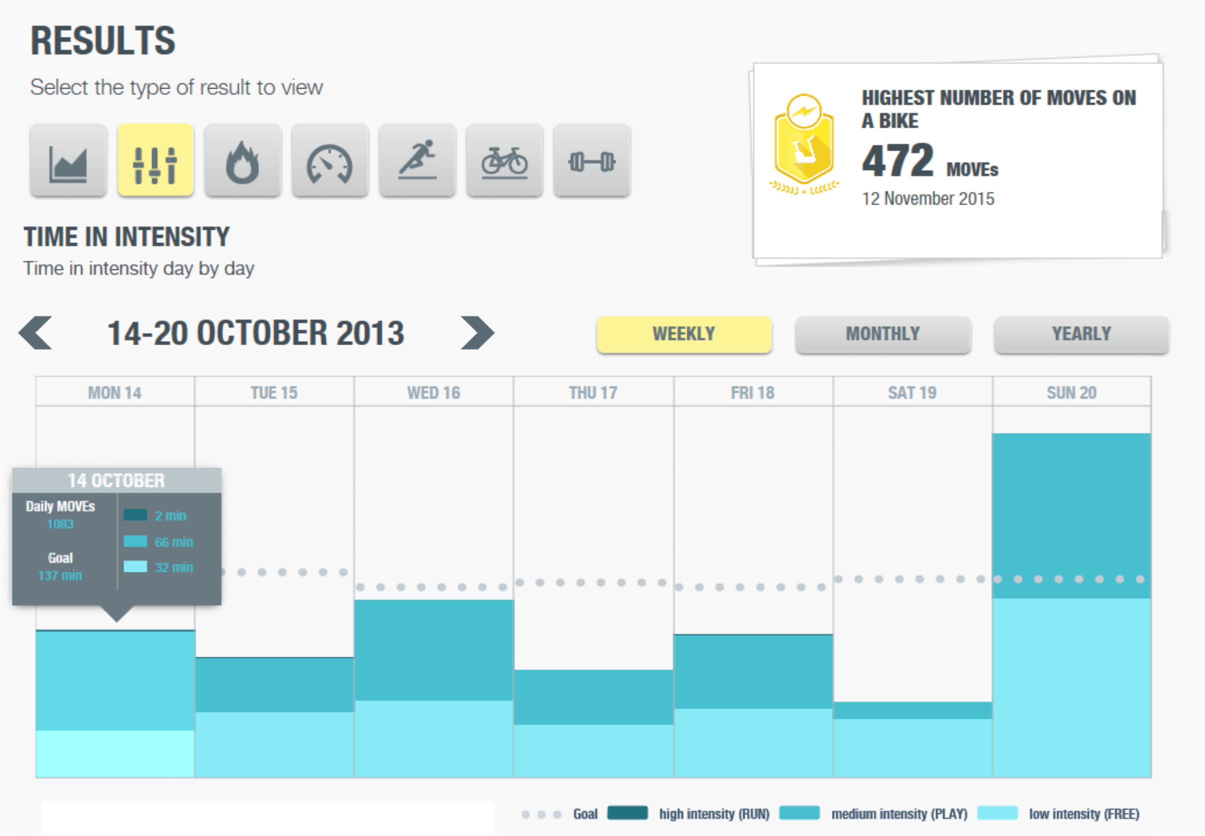
RESULTS
Select the type of result to view
TIME IN INTENSITY
Time in intensity day by day
HIGHEST NUMBER OF MOVES ON A BIKE
472 MOVEs
12 November 2015
14-20 OCTOBER 2013
WEEKLY
MONTHLY
YEARLY
MON 14
TUE 15
WED 16
THU 17
FRI 18
SAT 19
SUN 20
14 OCTOBER
Daily MOVEs
1083
Goal
137 min
2 min
66 min
32 min
Goal
high intensity (RUN)
medium intensity (PLAY)
low intensity (FREE)
