# Supplementary material for: Acceptability and Feasibility of Implementing Accelorometry-Based Activity Monitors and a Linked Web Portal in an Exercise Referral Scheme: Feasibility Randomized Controlled Trial
Source: J Med Internet Res. 2019 Mar 29;21(3):e12374. doi: 10.2196/12374 (PMC6460312; doi:10.2196/12374)
Supplement: Multimedia Appendix 2 [file jmir_v21i3e12374_app2.docx]

**Mean scores for usage and acceptability of intervention components.**

| Question | N | Mean (SD) | Possible range |
| --- | --- | --- | --- |
| **Acceptability and usage of the MyWellnessKey device** |  |  |  |
| How many times have you used the MWK in the last month? ^a^ | 49 | 0.4 (1.56) | 0 – 7 |
| How easy have you found the MWK activity monitor to use? ^b^ | 43 | 2.2 (1.45) | 0 – 4 |
| If you were to keep the MWK, how likely is it that you would continue to use it? ^c^ | 46 | 1.7 (1.58) | 0 – 4 |
| **Acceptability and usage of the MyWellnessCloud web portal** |  |  |  |
| How many times have you accessed the MWC in the last month? ^a^ | 47 | 0.3 (1.15) | 0 – 7 |
| How easy have you found the MWC website to use? ^b^ | 42 | 1.7 (1.31) | 0 – 4 |
| How likely is it that you will use the MWC website in the future? ^c^ | 46 | 1.1 (1.18) | 0 – 4 |

1. 0=Not at all, 1=Once in the last month, 2=Twice in the last month, 3=Three times in the last month, 4=Once a week, 5=Two or three days per week, 6=Four to six days per week, 7=Every day.
2. 0=Not at all, 1=Not very easy, 2=Neither easy or difficult, 3=Easy, 4=Very easy
3. 0=Not at all likely, 1=Unlikely, 2=Neither likely or unlikely, 3=Likely, 4=Very likely
