# Supplementary material for: Acceptability and Feasibility of Implementing Accelorometry-Based Activity Monitors and a Linked Web Portal in an Exercise Referral Scheme: Feasibility Randomized Controlled Trial
Source: J Med Internet Res. 2019 Mar 29;21(3):e12374. doi: 10.2196/12374 (PMC6460312; doi:10.2196/12374)
Supplement: Multimedia Appendix 3 [file jmir_v21i3e12374_app3.docx]

**Frequencies and percentage of missing data for whole sample at each time point.**

| Question and *data source* | Baseline (T0)  (N = 156) | | 16 week follow up (T1)  (N = 85) | | 52 follow up (T2)  (N = 105) | |
| --- | --- | --- | --- | --- | --- | --- |
|  | Missing data n (%) | | | | | |
| *Questionnaire data:* | Missing as participant did not answer question | All data missing from that specific time point | Missing as participant did not answer question | All data missing from that specific time point | Missing as participant did not answer question | All data missing from that specific time point |
| **Client Service Receipt Inventory** |  |  |  |  |  |  |
| Contacts with GP | 2 (1%) | 0 (0%) | 0 (0%) | 0 (0%) | 0 (0%) | 0 (0%) |
| Contacts with Nurse | 0 (0%) | 0 (0%) | 0 (0%) | 0 (0%) | 0 (0%) | 0 (0%) |
| Contacts with Healthcare Assistant | 0 (0%) | 0 (0%) | 1 (1%) | 0 (0%) | 1 (1%) | 0 (0%) |
| Contacts with Mental Health Professional | 0 (0%) | 0 (0%) | 1 (1%) | 0 (0%) | 0 (0%) | 0 (0%) |
| Contacts with Other Healthcare Professional | 1 (0.6%) | 0 (0%) | 8 (9%) | 0 (0%) | 8 (8%) | 1 (1%) |
| Contacts with Hospital Consultant | 4 (3%) | 0 (0%) | 2 (2%) | 0 (0%) | 1 (1%) | 1 (1%) |
| Contacts with Specialist Nurse | 0 (0%) | 0 (0%) | 2 (2%) | 0 (0%) | 2 (2%) | 1 (1%) |
| Contacts with Physiotherapist or Occupational Therapist | 1 (0.6%) | 2 (1%) | 2 (2%) | 0 (0%) | 4 (4%) | 1 (1%) |
| Contacts with Casualty (A&E department) | 2 (1%) | 0 (0%) | 1 (1%) | 0 (0%) | 2 (2%) | 1 (1%) |
| Contacts with Day Surgery Department | 0 (0%) | 0 (0%) | 1 (1%) | 0 (0%) | 3 (3%) | 1 (1%) |
| Overnight stay in Hospital | 0 (0%) | 0 (0%) | 0 (0%) | 0 (0%) | 2 (2%) | 1 (1%) |
| Contacts with Other Hospital Services | 1 (0.6%) | 0 (0%) | 7 (8%) | 0 (0%) | 3 (3%) | 1 (1%) |
| **Productivity losses** (collected only at T2) |  |  |  |  |  |  |
| Over the last 12 months how many days have you been unable to perform your usual activities? |  |  |  |  | 9 (9%) | 1 (1%) |
| Reason unable to perform usual activities |  |  |  |  | 18 (17%) | 1 (1%) |
| Has your work status changed in the last 12 months? |  |  |  |  | 5 (5%) | 1 (1%) |
| Reason for change in work status |  |  |  |  | 0 (0%) | 1 (1%) |
| If you are currently working, how many days off work due to ill health have you had in the last 12 months? |  |  |  |  | 19 (18%) | 1 (1%) |
| Please estimate how much your income has reduced by in the last 12 months |  |  |  |  | 9 (9%) | 1 (1%) |
| Please estimate how much your income has increased by in the last 12 months |  |  |  |  | 11 (10%) | 1 (1%) |
| Please state if there has been no change to your income |  |  |  |  | 8 (8%) | 1 (1%) |
| *Routinely collected data:* | | | | | | |
| **EQ-5D (5L)** |  |  |  |  |  |  |
| EQ-5D Utility Score | 27 (17%) | 22 (14%) | 19 (22%) | 23 (27%) | 19 (18%) | 63 (60%) |
| EQ-5D VAS Score | 25 (16%) | 22 (14%) | 19 (22%) | 23 (27%) | 19 (18%) | 63 (60%) |
| **Routinely collected contacts with GP** |  |  |  |  |  |  |
| GP Visits | 0 (0%) | 22 (14%) | 0 (0%) | 23 (27%) | 0 (0%) | 63 (60%) |
