# Supplementary material for: Acceptability and Feasibility of Implementing Accelorometry-Based Activity Monitors and a Linked Web Portal in an Exercise Referral Scheme: Feasibility Randomized Controlled Trial
Source: J Med Internet Res. 2019 Mar 29;21(3):e12374. doi: 10.2196/12374 (PMC6460312; doi:10.2196/12374)
Supplement: Multimedia Appendix 4 [file jmir_v21i3e12374_app4.docx]

Costs of delivering NERS with MWK as part of the feasibility trial varying unit cost of the MWK device^†^

| Annual NERS operational costs 2016-17 | Total (£) |
| --- | --- |
| *National costs paid by Welsh Government* | |
| Consultant | 2,384 |
| Physical Activity Specialist (Grade 8a) | 10,684 |
| Administrative support | 2,530 |
| Health Improvement coordinator | 1,392 |
| Meeting costs | 300 |
| Exercise Professionals (91.5 WTE) | 2,631,385 |
| Coordination and Office costs (e.g. printing, stationary) for all 22 local authorities | 71,848 |
| Training | 64,495 |
| Travel | 80,547 |
| *Joint national and local costs* | |
| Co-ordinator salary (23 WTE) Funding is split between local authorities (£368,438) and Welsh Government (£478,319) | 846,757 |
| *Local authority costs* | |
| Staff management | 75,000 |
| Promotional material | 22,000 |
| Room hire (no charge as covered by session costs) | 0 |
| Attending conferences | 2,200 |
| Total NERS annual operating costs (without MWK) | 3,811,522 |
| Participants in NERS^1^ | 15,626 |
| **Cost per participant** | **244** |
| *Additional costs related to MWK* | |
| Cost of MWK activity monitor device (based on 88 units purchased for the trial intervention group) | 7,920 (£90 per monitor x 88) |
| Cost of My Wellness Cloud annual licence fee (online professional web cloud) including VAT | 3,360 |
| Total MWK operating costs | 11,280 |
| Participants in receipt of MWK as part of the trial | 88 |
| Cost per participant for MWK | 128 |
| **Total cost per participants for NERS with MWK^2^** | **352** |

^†^ Costs rounded to the nearest pound (£).

^1^ Participants in NERS based on 15,470 individuals who took up the NERS programme September 2016 – August 2017 plus the 156 participants taking part in the trial (intervention n = 88, control n = 68)

^2^ Calculation – total annual operational cost per participant plus total cost per participant for MWK
